# Evaluation of Selected Honey and One of Its Phenolic Constituent Eugenol against L1210 Lymphoid Leukemia

**DOI:** 10.1155/2014/912051

**Published:** 2014-11-23

**Authors:** Saravana Kumar Jaganathan, Dilip Mondhe, Z. A. Wani, Eko Supriyanto

**Affiliations:** ^1^IJN-UTM Cardiovascular Engineering Centre, Faculty of Biosciences and Medical Engineering, Universiti Teknologi Malaysia, 81310 Skudai, Johor, Malaysia; ^2^Indian Institute of Integrative Medicine, Jammu, Jammu and Kashmir 180 001, India

## Abstract

People affected with leukemia are on the rise and several strategies were employed to thwart this deadly disease. Recent decade of research focuses on phenolic constituents as a tool for combating various inflammatory, cancer, and cardiac diseases. Our research showed honey and its phenolic constituents as crusaders against cancer. In this work, we explored the antileukemic activity of selected honey and one of its phenolic constituent eugenol against L1210 leukemia animal model. Results of this experiment showed that the selected honey samples as well as eugenol after intraperitoneal injection could not increase the median survival time (MST) of animals. Further, there was only slight marginal increase in the %*T/C* values of honey and eugenol treated groups. The number of phenolics present in the honey may not be a prime factor to promote antileukemic effect since there was no difference in the MST of two different honeys tested. This study limits the use of selected honey and eugenol against leukemia animal model.

## 1. Introduction

Leukemia is widely called blood cancer and it is characterized by uncontrolled growth of blood cells produced in the bone morrow. It has estimated that 6020 people (3,140 in males and 2,880 in females) will be affected and 1440 people will die of leukemia in 2014 [1]. This alarming rate necessitates the search for novel antileukemic agents gaining momentum.

Recent research from our laboratory showed honey containing higher phenolic constituents is potent in inhibiting colon cancer cells [2–4]. Also one of our recent works deciphered the apoptotic mechanism of eugenol against colon cancer cells [5, 6]. To further advance our research, we screened the* in vivo* antitumour efficiency of two selected honeys (Sample C possessing higher phenolic content (65.06% gallic acid equivalent (GAE)) and Sample B with lower phenolic content (29.96% GAE) [3] and eugenol against L1210 lymphoid leukemia animal model.

L1210 are derived from mouse lymphocytic leukemia cells originating from the ascitic fluid of 8-month-old female mice. Although it is lymphocytic B-cells they appear more like lymphoblasts in morphology. These cells are widely used in cytotoxic assays of chemotherapeutic agents. Kim et al., 1994, showed adenosine resulted in apoptosis of L1210 cells. Further, they showed apoptosis process was accompanied by distinct morphological changes including chromatin condensation and blebbing of plasma membranes [7]. Frequently, these cells are also injected in animals to develop an* in vivo* animal model bearing lymphocytic leukemia cells. This* in vivo* animal model appears to be a putative tool for investigating the antileukemic effect of anticancer compounds. Some of the studies used this model for studying antileukemic activity of N4-alkyl-1-beta-D-arabinofuranosyl cytosines, epirubicin, and so forth [8, 9].

This work reports the results of antitumor effects of the selected honey samples and eugenol against L1210 lymphoid leukemia animal model. This work will determine whether the number of phenolic constituents will have any effect on the antitumor activity of honey against lymphoid leukemia animal model.

## 2. Materials and Methods

### 2.1. Animals Used

 Model: L1210 lymphoid leukemia
 Sample: Sample C, Sample B, and eugenol
 Animals: CDF1
 Sex: male
 Weight: 18–23 g.

### 2.2. Experimental Method

L1210 lymphoid leukemia cells grown in the peritoneal cavity of DBA/2 female mice were collected from the animal harboring 6-7-day-old ascites. For testing, CDF1 males were used. 5 × 10^5^ cells were injected intraperitoneally in 30 CDF1 males weighing 18–23 g on day 0. The next day, animals were randomized and divided into five groups, containing 6 animals each. Groups I, II, and III were treated with Sample C, Sample B, and eugenol at the doses of 50% v/v (i.p.), 50% v/v (i.p.), and 100 mg/kg (i.p.), respectively, for 9 consecutive days. Group IV was treated with 5-fluorouracil (20 mg/kg) (i.p.) and it served as positive control. The control group received 0.2 mL normal saline (i.p.) for 9 consecutive days. The animals in each group were observed for mortality up to day 18 and the median survival time of animals in each group was calculated. The antileukemic activity was assessed by use of the criterion %*T/C*, where* T* was the median survival time (MST, days) of the drug treated mice, bearing L1210 lymphoid leukemia, and* C* was the median survival time (MST, days) of untreated control animals, bearing the same leukemia.

## 3. Results 

Results obtained for the* in vivo* antitumour activity of selected honey samples and eugenol were listed in Table 1. From the results, it can be inferred that median survival time (MST) of all the honey samples tested did not show any significant differences compared to the positive control. %*T/C* values estimated for 5-FU were 166.6, whereas for the honey samples they were found to be 102.56, 104.61 for Sample B and Sample C, respectively. For eugenol, they were found to be 110.25.

## 4. Discussion

Observed results are similar to our recently published work which studied the effect of honey on Ehrlich solid carcinoma [10]. Although Sample C was found to be potent in inhibiting Ehrlich ascites, the preventive effect on Ehrlich solid carcinoma was diminutive at the range of 4% tumor inhibition. Phenolic content of Sample C and Sample B was 65.06 Gallic acid equivalent (GAE) and 29.96 GAE, respectively [3]. Inspite of their significant difference in their phenolic content, %*T/C* values show barely discernible difference between the two honey samples. Researchers had identified some phenolic components of honey that have been attributed to the antileukemic activity [11]. Hence, we assume that qualitative nature of phenolic constituents present in the honey may play a vital role against the leukemia but not the total phenolic content of honey.

Previous work from our laboratory showed eugenol at a dose of 100 mg/kg i./p. was able to inhibit the growth of Ehrlich ascites by 28.88%. Moreover, it has been found that eugenol (100 mg/kg; i./p.) showed 24.35% tumor growth inhibition of Ehrlich solid carcinoma. However, our results with leukemia animal model are not promising. Although the estimated %*T/C* and MST were higher for eugenol compared to honey samples, they were not significant compared with the positive control. However, eugenol has shown to be effective in inducing apoptosis in HL-60 leukemia cells through reactive oxygen species (ROS) generation [12]. Hence, it would be interesting for further studies to document the effect of eugenol on HL-60 bearing animal model to further validate eugenol as a possible candidate against leukemia.

In summary, this work evaluated the antitumor effect of honey samples and eugenol against L1210 leukemia animal model. Result showed both honey samples and eugenol displayed nonsignificant antileukemic activity compared with the positive control. This result serves as an indicator that the higher phenolic content of honey may not be always a prime factor to promote antitumor effect in leukemia animal model. Further apoptotic activity of eugenol in certain leukemia cancer cell lines may not correlate with the antileukemic effect observed on L1210 animal model.

## Figures and Tables

**Table 1:** Antileukemic activity of selected honey and eugenol.

Treatment groups	Median survival time	% *T*/*C*	Inference
Sample B (50% v/v i.p.)	8.0 days	102.56	Nonsignificant activity
Sample C (50% v/v)	8.16 days	104.61	Nonsignificant activity
Eugenol (100 mg/kg i.p.)	8.6 days	110.25	Nonsignificant activity
5-FU (20 mg/kg i.p.)	13.0 days	166.66	Significant activity
Control (NS 0.2 mL i.p.)	7.8 days		
